# Liver Fibrosis Conventional and Molecular Imaging Diagnosis Update

**Published:** 2019-01-22

**Authors:** Shujing Li, Xicui Sun, Minjie Chen, Zhekang Ying, Yamin Wan, Liya Pi, Bin Ren, Qi Cao

**Affiliations:** 1 Department of Diagnostic Radiology and Nuclear Medicine, University of Maryland School of Medicine, Baltimore, Maryland, USA; 2 Department of Radiology, The first affiliated Hospital of Hebei Medical University, Shijiazhuang, Hebei province, P.R.China; 3 Department of Medicine, University of Maryland School of Medicine, Baltimore, Maryland, USA; 4 Department of Radiology, the First Affiliated Hospital of Zhengzhou University, Zhengzhou, Henan province, P.R.China; 5 Department of Pediatrics in the College of Medicine, University of Florida, Gainesville, Florida, USA; 6 Department of Surgery, University of Alabama at Birmingham School of Medicine, Alabama, USA

**Keywords:** Liver fibrosis, Nuclear medicine, Magnetic resonance imaging, Ultrasound, Computed tomography

## Abstract

Liver fibrosis is a serious, life-threatening disease with high morbidity and mortality that result from diverse causes. Liver biopsy, considered the “gold standard” to diagnose, grade, and stage liver fibrosis, has limitations in terms of invasiveness, cost, sampling variability, inter-observer variability, and the dynamic process of fibrosis. Compelling evidence has demonstrated that all stages of fibrosis are reversible if the injury is removed. There is a clear need for safe, effective, and reliable non-invasive assessment modalities to determine liver fibrosis in order to manage it precisely in personalized medicine. However, conventional imaging methods used to assess morphological and structural changes related to liver fibrosis, including ultrasound, computed tomography (CT), and magnetic resonance imaging (MRI), are only useful in assessing advanced liver disease, including cirrhosis. Functional imaging techniques, including MR elastography (MRE), US elastography, and CT perfusion are useful for assessing moderate to advanced liver fibrosis. MRE is considered the most accurate noninvasive imaging technique, and US elastography is currently the most widely used noninvasive means. However, these modalities are less accurate in early-stage liver fibrosis and some factors affect the accuracy of these techniques. Molecular imaging is a target-specific imaging mechanism that has the potential to accurately diagnose early-stage liver fibrosis. We provide an overview of recent advances in molecular imaging for the diagnosis and staging of liver fibrosis which will enable clinicians to monitor the progression of disease and potentially reverse liver fibrosis. We compare the promising technologies with conventional and functional imaging and assess the utility of molecular imaging in precision and personalized clinical medicine in the early stages of liver fibrosis.

## Introduction

Liver fibrosis is a common pathological consequence of chronic liver disease, including chronic infections by hepatotropic viruses (hepatitis B and hepatitis C viruses) [1,2], chronic exposure to alcohol[3], chronic metabolic alterations, (e.g., non-alcoholic fatty liver disease [4], autoimmune causes, (e.g., autoimmune hepatitis) [5], and the relative chronic activation of the wound-healing reaction [6]. Liver fibrosis is characterized by the excessive accumulation of extracellular matrix (ECM) components including collagen fibers that occur in most types of chronic liver diseases. These and non-collagenous components of liver fibrosis often lead to hepatic dysfunction, portal hypertension, cirrhosis, and even hepatocellular carcinoma [7]. Liver fibrosis is a dynamic process of hepatic homeostasis mediated by several cellular mediators in response to an inflammatory process. Hepatic stellate cells (HSCs) are a key component in liver fibrosis. In the fibrogenic liver, quiescent HSCs transdifferentiate into proliferative, migratory, and contractile myofibroblasts, manifesting pro-fibrogenic transcriptional and secretory properties (so-called “cell activation”), and secrete ECM molecules that accumulate and form scar tissue in the space of Disse that leads to sinusoidal capillarization, characterized by loss of endothelial fenestrations [8]. In terms of the molecular pathway, many studies have focused on HSC/myofibroblasts because of their perpetuated activation through multiple signal pathways, including membrane receptor signaling pathway [e.g. tumor growth factor beta (TGF-β)] [9], platelet-derived growth factor (PDGF) [10], toll-like receptor (TLR) [11] Wnt/β-catenin signaling[12]), nuclear receptor signal pathway [e.g. farnesoid-X-receptor (FXR)] [13], peroxisome proliferator-activated receptor (PPAR) [14 PMID:], transcription factor [e.g. sex-determining region Y-box (SOX9)] [15], and myocardin-related transcription factor A (MRTF-A)[16].

Both invasive and non-invasive tools for evaluating liver fibrosis are available. Liver biopsy, an invasive method, is considered the “gold standard” to diagnose, grade and stage liver fibrosis. In 1981, Knodell et al. established the first semiquantitative and reproducible histology scoring system, the Histology Activity Index (HAI) [17]. However, the METAVIR scoring system (1996) by Bedossa et al. [18] is currently the most popular, and comprises five stages: F0 (no fibrosis), F1 (minimal fibrosis, portal fibrosis without septa), F2 (moderate fibrosis, portal fibrosis with few septa), F3 (severe fibrosis, sepal fibrosis with many septa but no cirrhosis), F4 (cirrhosis). However, liver biopsy has some limitations in accurately defining liver disease and evaluating its progress, including invasiveness, cost, morbidity, mortality [19] tumor seeding [20], sampling variability [21], inter-observer variability [22] and the dynamic process of fibrosis [23]. Conventionally, liver fibrosis has been considered potentially reversible, while cirrhosis as the end-stage of the pathological process has been considered irreversible. However, compelling evidence from animal models and human studies has demonstrated if the injury is removed, all stages of fibrosis are reversible [24–26]. There is a clear need for safe, effective, and reliable non-invasive assessment modalities to determine liver fibrosis in order to precisely manage it in personalized clinical medicine. Imaging methods including ultrasound (US), computed tomography (CT), magnetic resonance imaging (MRI), and nuclear molecular imaging, have the potential to provide significant benefits in clinical diagnosis, management, and treatment monitoring. This review intends to provide an overview of recent advancements in molecular imaging for the diagnosis and staging of liver fibrosis, and monitoring the progression and recovery of liver fibrosis. We also compare the promising technologies with conventional and functional imaging.

A summary of latest advances in conventional and molecular imaging is listed in the Table 1, which would be convenience for readers to catch up the latest imaging knowledge.

## Evaluation of Liver Fibrosis by MRI, US, and CT

### MRI

#### Conventional MRI:

Conventional MRI can evaluate morphologic and structural changes related to liver fibrosis. Imaging features include surface nodularity [27], widening of fissures, expanded gallbladder fosse sign, posterior hepatic notch sign, increased caudate to right lobe ratio [28], enlargement of the lateral segments of the left lobe and caudate lobe [29], regenerative nodules [30], splenomegaly, porto-systemic varices, ascites, and bowel wall thickening, and peak enhancement at the late phases (venous/equilibrium phases) [31]. However, in terms of diagnosis of early-stage of liver fibrosis, the technique is less sensitive.

### Functional MRI

#### Susceptibility-weighted MRI (SWI):

SWI is a gradient echo sequence with increased sensitivity to the presence of iron, hemoglobin, and calcifications. SWI has a demonstrated ability to quantify and grade liver fibrosis in patients with chronic liver disease. However, it is more sensitive in high-stage liver fibrosis (F3, F4) than low-stage liver fibrosis (F0–F2) because of more nonhomogeneous iron deposition and secondary pathologic changes that occur at higher grades of liver fibrosis [32].

#### Diffusion weighted MRI (DWI):

Reports differ regarding DWI’s ability to quantify liver fibrosis in chronic liver disease. Some studies concluded that DWI could be considered a valid non-invasive method to predict the presence of moderate or advanced fibrosis [33]. Other studies have investigated the use of ADC in distinguishing different stages of liver fibrosis [34,35]. There are limitations of DWI in the assessment of liver fibrosis, such as the fact that diffusion is affected by perfusion changes, hepatic steatosis, the presence of iron in the tissue, and inflammatory changes [36]. Because of these factors, DWI is not considered reliable and sensitive enough to distinguish early-stage liver fibrosis.

#### Perfusion MRI (PWI):

PWI is based on dynamic contrast-enhanced MRI and can be used to quantify the microcirculatory status of the liver parenchyma. The deposition of collagen seen in liver fibrosis causes the gradual obliteration of intrahepatic vessels and sinusoids and slows the passage of blood within the parenchyma which leads to a decrease in portal venous flow to the liver, an increase in hepatic arterial flow, and the subsequent formation of intrahepatic shunts [31]. These kinetic flow changes related to liver fibrosis can be assessed with PWI [37]. Dynamic gadolinium ethoxybenzyl diethylenetriaminepentaacetic acid (Gd-EOB-DTPA) enhanced MRI was shown to be reliable in the staging of liver fibrosis [38–40]. However, perfusion is also affected by other factors, such as cardiac status, hepatic portal venous flow, and hepatic congestion, etc.

#### MR elastography (MRE):

Several studies have shown that MRE can measure the stiffness of the liver associated with fibrosis and is useful and reliable in assessing the pathological grades of liver fibrosis [41,42] even in pediatric patients [43]. MRE has a lower accuracy level in assessing mild stages of (F0, F1) liver fibrosis [44,45] and there are some factors such as inflammation [46] and cardiac function [47] that can affect the mean liver stiffness assessed by MRE. However, the method currently is considered the most accurate noninvasive imaging technique for assessment of moderate to advanced (F2–4) liver fibrosis[48]. Morisaka and colleagues compared the liver fibrosis stages of 80 patients by 2D liver MRE with gradient-echo based sequence on a 1.5 or 3.0T scanner with 120 patients who underwent liver biopsy. They concluded that MRE is as accurate as liver biopsy for liver staging. The other merit of the technique is perfect interobserver agreement [49].

#### T1 mapping MRI:

It has been shown that T1 mapping on Gd-EOB-DTPA-enhanced or gadoxetic acid-enhanced MRI may be a reliable diagnostic tool in the staging of liver fibrosis in animal and human models [50–53]. Sheng and colleagues analyzed the parameters of different stages of liver fibrosis using T1 mapping Gd-EOB-DTPA-enhanced MRI, and found that there were strong correlations between liver fibrosis and hepatobiliary phase T1 relaxation time (r=0.960) and reduction rate (r=−0.952). They also found that fibrosis was the only independent predicted parameter by multivariate analyses [50]. However, T1 mapping approaches are often confounded by the extracellular space contrast-enhancement effect of gadoxietic acid in liver cirrhosis [54].

#### MRI texture analysis:

Texture analysis can assess the changes in the texture of live parenchyma associated with fibrosis [30], and the imaging can be acquired with a variety of sequences, including non-contrast [55], contrast-enhanced [56], and even double contrast enhanced imaging [57]. Texture analysis on MR imaging has the ability to stage and quantitate liver fibrosis in animal models [55,58] and can assess the stage of hepatic fibrosis of moderate to advanced liver fibrosis, but is less sensitive for staging mild levels of fibrosis in patients [56,59].

#### Spin-lattice relaxation time in the rotating frame (T1ρ) MR Imaging:

T1ρ refers to a phenomenon that occurs when tipping the magnetization of spins into the transverse plane before applying a radiofrequency pulse creates a spin-lock state, leading to a low-frequency precession [60]. T1ρ can reflect biologic processes associated with alterations in macromolecular composition and proton exchange in tissues. There are contradictory results about the role of T1ρ MR imaging in staging liver fibrosis. Takayama and colleagues found that T1ρ relaxation was not significantly correlated with liver fibrosis (p=0.95) in patients with chronic liver disease [61]. Several studies have shown that T1ρ is useful in evaluating the stage of liver fibrosis. Wang and colleagues reported that T1ρ imaging is able to detect early liver fibrosis in a rat biliary duct ligation model [62]. Li and colleagues found that T1ρ was as good as ultrasonography elastography for detecting and staging liver fibrosis in rabbit models (r=0.693) [63]. Singh and colleagues reported that T1ρ values in fibrotic livers were significantly higher than those of healthy livers and there was significant correlation between stages of liver fibrosis and T1ρ values (r=0.99, P<0.05) [64]. Further studies are required to assess the diagnostic accuracy of T1ρ MR imaging for staging of liver fibrosis and to compare this technique to MRE and liver biopsy.

### Ultrasonography (US)

#### Conventional US:

Conventional US is a useful technique to assess morphological and structural changes to the liver, and as such is useful in evaluating cirrhosis. However, it is not sensitive for evaluating and staging early fibrosis. Some studies reported that conventional US might overestimate the role of liver fibrosis [65]. D’Onofrio and colleagues reported conventional US has a sensitivity of only 25% in identifying liver fibrosis in chronic liver disease [66].

#### Contrast-enhanced US:

Contrast-enhanced US has been considered useful for assessing different stages of liver fibrosis by evaluating hemodynamic alteration. Qiu and colleagues found that portal vein maximum signal intensity was accurate in diagnosing fibrosis stages, especially non-advanced fibrosis stages (≥F1,F2). However, the accuracy of contrast-enhanced US was still lower than US elastography[67].

#### US elastography:

US elastography is the most widely used imaging method for the evaluation of liver fibrosis in clinical practice; and the technique is widely recognized as reliable and easy to perform at the point of care. There are several types of US elastography: strain elastography (SE), [also called real-time tissue elastography (RTE)], transient elastography (TE), acoustic radiation force imaging (ARFI), and shear wave elastography (SWE).

#### SE:

SE is a semi-quantitative method based on the ratio strain between two regions of interest and the stiffness is indicated in the color scale using conventional US equipment. SE is regarded as a promising technique capable of noninvasively evaluating and staging liver fibrosis with high diagnostic accuracy. Meng and colleagues performed SE and liver biopsy in 166 patients with chronic hepatitis B, and they found the diagnostic accuracy of SE was similar to that of TE; the elasticity index and liver stiffness were 0.880 and 0.909 (≥F2), 0,868 and 0.874 (≥F3), and 0.752 and 0.815 (F4), respectively for predicting substantial fibrosis [68]. A study was conducted by Tajiri and colleagues using SE in 598 patients with chronic liver disease and they found that the elasticity index was significantly different between mild (F1–2) and advanced fibrosis (F3–4) [69].

#### TE (Fibroscan):

TE was the first US-based elastographic method to evaluate elasticity by measuring the velocity of elastic shear waves in parenchyma generated by a mechanical push. The first clinical data of hepatic fibrosis using this technique were published in 2003 [70]. Many studies have shown that TE is an effective noninvasive tool for assessing and staging liver fibrosis. In a meta-analysis by Li and colleagues, the sensitivity of SE in chronic hepatitis B patients for staging liver fibrosis F≥2, F≥3 and F≥4 was 0.806, 0.819, 0.863, the specificity was 0.824, 0.866, 0.875, and the receiver operating characteristic curve was 0.88, 0.91, 0.93, respectively [71]. Sharma et al founded that there was a significant difference in liver stiffness measurements in patients with stage F0 compared with patients with F1+F2 (4.5 vs. 7.5 kpa, P=0.001) and F3+F4 (4.5 vs. 19.4 kpa, P=0.001)[72].

#### Acoustic radiation force impulse (ARFI):

AFRI is a US-based elastographic method that evaluates elasticity by measuring tissue displacement in a region of interest that has been excited with acoustic pulse shear waves. The first clinical data of assessing hepatic fibrosis using this technique were published in 2009 [73]. ARFI is considered a reliable noninvasive tool for assessing and staging liver fibrosis. There has been some controversy over whether ARFI or SE provides better diagnostic value. A study by Ragazzo and colleagues with 107 chronic hepatitis C patients found that the accuracy of SE and ARFI for staging liver fibrosis F0–F1, F1–F2, F2–F3, and F3–F4 were 0.81and 0.78, 0.73 and 0.53, 0.70 and 0.64, 0.98 and 0.96, respectively [74], which indicated that SE was more effective than AFRI. However, López et al. concluded that ARFI was more cost-effective as a first line technique for staging liver fibrosis, with accuracy similar to SE [75].

#### SWE:

SWE is a novel, real-time, two-dimensional elastography technique, which can accurately assess the stage of liver fibrosis by estimating stiffness quantitatively in kilopascals. The first clinical data of hepatic fibrosis using this technique were published in 2012 [76]. In a meta-analysis by Li and colleagues, the sensitivity of SWE for staging liver fibrosis F≥2, F≥3 and F≥4 was 0.85, 0.90, 0.87, the specificity was0.81, 0.81, 0.88, and the receiver operating characteristic curve was0.88, 0.94, 0.92, respectively. They concluded that the accuracy of SWE was similar to ARFI, but more accurate than RTE and TE for staging liver fibrosis CT [77]. A more recent study by Gao et al. in 402 patients with chronic hepatitis B found that the areas under the receiver operating characteristic curve of two-dimensional SWE (0.87) were higher than that of TE (0.80) and ARFI (0.70) [78].

It is not surprising then that US elastography is a promising technique for evaluating liver fibrosis because of its high accuracy and sensitivity to stages of moderate to severe liver fibrosis. However, these techniques are still not sensitive and accurate enough to differentiate among stages of mild fibrosis, especially between F0–1 and F2.

### Computed tomography(CT)

It is well-known that the morphological liver changes related to cirrhosis can bedetected accurately using CT, but CT is not considered to be sensitive enough for staging less advanced stages of liver fibrosis. However, several studies using artificial intelligence in conjunction with CT have shown that the modality can assess and stage liver fibrosis accurately.

#### Conventional CT:

Conventional CT is useful in assessing morphological features for the diagnosis of liver fibrosis and cirrhosis. Several studies have validated CT’s ability to stage liver fibrosis using quantitative morphological liver analysis. Huber et al. used a retrospective analysis of CT images of 148 patients and they found that the sensitivity and specificity of sum of liver vein diameters divided by the caudate-right lobe ratio <24 were 0.83 and 0.76 for staging F0–3[79]. Another study by Pickhardt et al performed hepatosplenic volume analysis in 624 patients by CT; they found that the liver segmental volume ratio (0.26 ± 0.06, 0.25 ± 0.08, 0.33 ± 0.12, 0.39 ±0.15, 0.56 ± 0.30, F0–4, respectively) and total splenic volumes (215.1 ±88.5mm3, 294.8 ± 153.4mm3, 291.6 ± 197.1mm3, 509.6 ± 402.6mm3, 790.7 ± 450.3mm3, F0–4, respectively) increased with the stage of fibrosis [80].

#### CT perfusion (CTP):

Several studies have shown that perfusion CT may help differentiate minimal fibrosis from intermediate fibrosis in patients with chronic liver disease and mean transient time was the most sensitive parameter for staging liver fibrosis [81]. A study by Wang et al. performed CTP in rabbit liver fibrosis models; they conducted that portal venous perfusion was the most promising of parametric perfusion indexes [82].

#### Fibro CT:

Fibro CT, also called weighted CT mean fibrosis, provides optical analysis of CT images of the liver utilizing conventional CT scan images coupled with additional software. This technique was used to detect the stage and distribution of liver fibrosis in patients with chronic hepatitis C [83].

#### CT texture analysis:

CT texture analysis is considered a reliable, non-invasive method to detect and assess the advanced stages of liver fibrosis. However, the technique is less sensitive in assessing mild liver fibrosis. A study by Lubner et al. evaluated CT texture analysis for staging liver fibrosis in 556 patients; they found that mean gray-level intensity increased with fibrosis stage. For significant fibrosis (F≥2), mean receiver operating characteristic area under the curve (AUC) was0.80, with sensitivity and specificity of 74% and 75% using a threshold for 0.44, for advanced fibrosis (F≥3), similar AUC and sensitivity/specificity were attained [84].

#### Deep learning-based algorithms:

A few recent studies have shown that deep learning-based algorithms allow for a highly accurate assessment of liver fibrosis and are considered to be a promising and widely applicable method for staging liver fibrosis. A study by Choi et al. used CT imaging, including portal venous phase CT images, in 891 patients with pathologically confirmed liver fibrosis. Using a deep learning system, they achieved a staging accuracy of 79.4% (707 of 891) and the area under the receiver operating characteristic curve of0.96, 0.97, and 0.95 for diagnosing significant fibrosis (F2–4), advanced fibrosis (F3–4) and cirrhosis (F4), respectively [85]. There is a large overlap of the different parameters of CT, and these techniques are not accurate or sensitive enough to stage early fibrosis.

## Evaluation of Liver Fibrosis using Molecular Imaging

As noted above, while conventional and functional imaging is useful for assessing and staging liver fibrosis, especially moderate to advanced liver fibrosis (F2–4), these techniques are less sensitive and accurate in detecting and quantifying early-stage (F0–1) liver fibrosis. Molecular imaging is used in the visualization, characterization, and measurement of biological process at the molecular and cellular levels in humans and other living systems [86]. Recent studies of molecular imaging, including nuclear imaging and molecular MRI of liver fibrosis, have been used for gaining molecular information by utilizing target-specific molecular probes that assess certain components of the ECM or HSCs in early-stage fibrotic livers. A number of molecules including collagen types I [87], III [88], and IV [89] are present in increased amounts in fibrotic livers. For example, the amount of type I collagen in fibrotic livers is increased from 36% to 53% compared to the normal liver [90]. The results of the studies using molecular imaging are exciting and the details are elaborated below.

### Nuclear medicine

#### (99m)Tc-3PRGD2 probe targeting integrin αvβ3:

Liver fibrogenesis is intimately associated with the activation of HSCs, and the fibrotic process is accompanied by the reduction of activated HSCs. During the process of liver fibrosis, integrin αvβ3 is highly expressed after activated HSCs are transformed to myofibroblasts [91]. Zhang et al. intravenously administered (99m)Tc-3PRGD2 in the livers of rats with thioacetamide-induced liver fibrosis. They found that the uptake and retention of (99m)Tc-3PRGD2 in the fibrotic liver enhanced compared with the control group, and the radiotracer bound specifically with the integrin αvβ3 mainly expressed on the activated HSCs [92]. Another study by Yu et al. using a rat model found the accumulation of (99m)Tc-3PRGD2 in the liver increased in proportion to the progression of fibrosis and extended with the exposure time to thioacetamide. They also found that as early as week 4 of injury, the accumulated levels were significantly different compared to the control group (liver-to-background ratio: 32.30 ± 3.39 vs. 19.01 ± 3.31; P=0.0002). The expression of integrin αvβ3 on the activated HSCs was demonstrated by ex vivo immunofluorescence staining and there was a strong correlation between the levels of integrin αvβ3 and PET/CT (R=0.75, P<0.001). They also assessed the recovery from liver fibrosis and found that (99m)Tc-3PRGD2 uptake in the fibrotic liver decreased after antibiotic therapy as compared to the control group [93]. (99m)Tc-3PRGD2 PET/CT is a promising technique in terms of specificity and accuracy of quantitating and staging liver fibrosis, including early stages of hepatic fibrosis. In addition, the technique is useful to monitor the progression and recovery of hepatic fibrosis.

#### (99m)Tc-GSA/(99m)Tc-p(VLA-co-VNI) probe targeting Asialoglycoprotein receptor (ASGP-R):

ASGP-R is expressed in the mammalian liver and located on the surface of hepatocytes’ membranes. (99m)Tc-GSA is an albumin ramification and has been used as an ASGP-R-binding radiopharmaceutical in clinical research in Japan since 1992 [94]. A study by Taniguchi et al. evaluated the clinical utility of hepatic clearance with (99m)Tc-GSA using SPECT in 78 patients with liver fibrosis and hepatic carcinoma and found that there was a negative correlation between hepatic clearance with (99m)Tc-GSA and the degree of liver fibrosis (R=−0.598, P<0.00001) and this parameter was a significant independent predictor of liver fibrosis [95]. Another study by Yoshida et al. compared quantitative indices using (99m)Tc-GSA SPECT in 161 patients with liver fibrosis and they found that the area under curve values of liver uptake value (LUV), and functional liver index (FLI) for predicting severe fibrosis were 0.73 and 0.83, respectively. The study showed 65% sensitivity, 88% specificity and 76% accuracy using an FLI value of 0.053 [96]. (99m)Tc-p(VLA-co-VNI) is another ASGP-R binding agent with excellent hepatic targeting and biological properties; it was synthesized by Liu et al. in 2014 [97]. A study by Zhang et al. using a carbon tetrachloride-induced mouse model demonstrated a decreased expression of ASGP-R by ex vivo Western blot analysis. (99m)Tc-p(VLA-co-VNI) specifically targeted ASRP-R in a competitive inhibition experiment. There was a strong negative correlation between LUV using SPECT/CT in vivo and liver hydroxyproline levels (R=−0.83). They also assessed the therapeutic efficacy of Tan IIA in treating liver fibrosis. Tan IIA is a potential drug for treatment of hepatic fibrosis. They found that (99m)Tc-p(VLA-co-VNI) uptake in fibrotic liver tissue increased after therapy compared with the control group [98].

#### 99mTc-CBP1495 (CPKESCNLFVLKD) probe targeting type I collagen:

A study by Zhang et al. identified that CBP1495 was an original collagen-binding peptide and could effectively bind to collagen type I(Kd=861nM) in vitro and ex vivo using Western blot and histochemistry analyses. They performed SPECT/CT imaging with 99mTc-CBP1495 in a rat fibrosis model and found that 99mTc-CBP1495 accumulated in fibrotic livers and there was a strong positive correlation between 99mTc-CBP1495 uptake and liver hydroxyproline levels (R=0.7581, P<0.0001) [99].

#### [18F]FEDACN-benzyl-N-methyl-2-[7,8-dihydro-7-(2-[18F]fluoroethyl)-8-oxo-2-phenyl-9H-purin-9-yl]-acetamide) probe targeting translocator protein (18kDa) (TSPO):

TSPO is expressed in HSCs, which are the major ECM-producing cells in liver fibrosis both in vitro and in vivo [100]. Hatori et al. intravenously administered [18F]FEDAC in the livers of rats with thioacetamide-induced liver fibrosis. They found that TSPO was expressed mainly in HSCs, the uptake of [18F]FEDAC in fibrotic livers was well correlated with TSPO expression, and TSPO mRNA levels increased with the level of liver fibrosis [101].

#### 99mTc-mebrofenin cholescintigraphy (99mTc-MHS) and 99mTc-red blood cells (99mTc-RBC) and liver fibrosis:

It has been shown that 99mTc-MHS and related compounds can assess liver function and 99mTc-MHS-binding is altered by the presence of liver disease. Kula et al. performed PET/CT on 62 patients with HCV using 99mTc-MHS. They found that the uptake rates of 99mTc-MHS were significantly decreased in mild, moderate, and severe liver fibrosis groups compared with controls (P<0.05) and the correlation between the severity of fibrosis and 99mTc-MHS uptake rate was strongly significant (r=0.81, P<0.0001). However, the time required for maximal hepatic activity (Tmax), and the time required for peak activity to decrease by 50% (T1/2max) were prolonged only in the moderate and severe liver fibrosis groups compared to the control group [102]. In a study by Papantonious et al., 24 patients with liver fibrosis were examined with PET/CT using 99mTc-MHS and 99mTc-RBC. They found that 99mTc-MHS could not differentiate fibrotic from normal parenchyma, whereas 99mTc-RBC could (P<0.001), and there were significant differences between the liver to heart (L/H) ratios and fibrotic and cirrhotic lesions in both modalities (99mTc-MHS: P=0.024, 99mTc-RBC: P=0.003) [103].

#### ^13^N-NH3·H2O PET/CT and liver fibrosis:

The traditional view is that water, ^3^N-NH3·H2O (ammonia), urea, and other small molecules pass through the cell membrane by free diffusion. However, there are differing theories, one known as aquaporin (AQP) theory, explains the mechanism of water transport across the cell membrane through aquaporins [104]. Aquaporins are specific transporters of the water protein family that can significantly increase cell membrane permeability and can regulate the balance of water inside and outside cells. AQP1, AQP3, AQP4, AQP7, AQP8, AQP9 and AQP11 are distributed in normal liver cells [105]. The number of liver cells and the function and distribution of liver tissues are changed during the pathological process of liver fibrosis which makes it possible to detect and stage of hepatic fibrosis. Han et al. performed ^13^N-NH3 H2O PET/CT in rat model and they found that the mean standard uptake value (SUVmean) increased in early stage fibrosis (20s 1.2 ± 0.8) compared to the control group (0.72 ± 0.23) [106]. ^13^N-NH3·H2O PET/CT may be a new method for the diagnosis and staging of liver fibrosis, but further studies are needed.

#### [18F]fluoro-proline and liver fibrosis:

There are four isomers of 4-[18F]fluoro-proline, cis-4-[18F]fluoro-L-proline trans-4-[18F]fluoro-L-proline, Cis-4-[18F]fluoro-D-proline, trans-4-[18F]fluoro-D-proline, and all have discrete physiological behaviors. In a whole body distribution study, Cis-4-[18F]fluoro-L-proline showed high retention in the renal cortex and slight uptake in the liver and pancreas in a study of six patients [107]. Trans-4-[18F]fluoro-L-proline exhibited no retention in the renal cortex, liver, and pancreas but an increased uptake in soft tissue and muscles in another human study [108], Cis-4-[18F]fluoro-D-proline showed a considerable amount of uptake in the urinary tract and slight uptake in the skeleton, and trans-4-[18F]fluoro-D-proline exhibited uptake in the urinary tract and high uptake in the skeleton [109]. The studies using 4-[18F]fluoro-L-proline are mainly focused on the brain [110], the urologic system [111], pulmonary fibrosis [112] and the musculoskeletal system [113]. The capability and role of 4-[18F]fluoro-proline in collagen synthesis has been investigated by serval studies. It has shown that excessive scar formation is accompanied by abnormal collagen synthesis. However, in terms of evaluating collagen synthesis by studying the uptake of cis-4-[18F]fluoro-L-proline labeled in PET imaging, there is controversy. An in vivo in rat model study concluded that the tracer is not suitable for monitoring collagen synthesis in scar formation [114]. Two PET studies using cis-4-[18F]fluoro-L-proline in rabbit models with lung fibrosis indicated uptake of the tracer at the early stage of disease [115,116]. However, in an idiopathic pulmonary fibrosis study of five patients, low pulmonary uptake of cis-4-[18F]fluoro-L-proline was observed [112]. The slow nature of fibrogenesis, the relative low dose of the tracer, or no participation of this tracer in collagen synthesis may be possible explanations for this. Results of 4-[18F]fluoro-proline in liver fibrosis, which is characterized by the excessive accumulation of ECM components (including collagen fibers and non-collagenous components) have not been reported.

However, 4-[18F]fluoro-proline, especially cis-4-[18F]fluoro-L-proline, can be used as a tracer using PET to assess liver fibrosis.

## Molecular MRI

The molecular MR imaging of liver fibrosis is based on the use of contrast agents. In general, there are two types of contrast agents: paramagnetic compounds, also called T1 contrast agents, which usually are composed of Gadolinum^3+^ or Mn^2+^ and superparamagnetic compounds, also called T2 contrast agents, which often are constructed with iron oxide [117].

### Gadolinium-diethylenetriamine pentaacetic acid-GKWHCTTKFPHHYCLY (EP-3533)/CM-101 probe targeting type I collagen

#### EP-3533 probe:

EP-3533 is a type I collagen-targeting T1 MR contrast agent which has been demonstrated to be useful for diagnosing and staging liver fibrosis in animal studies [118]. Fuchs et al. utilized EP-3533 to target type I collagen to stage liver fibrosis in a carbon tetrachloride mouse model. They found that the technique was more sensitive than T1/T2 relaxation time of MRI, or the ADC value of DWI. The most sensitive biomarker was the liver muscle contrast to noise ratio which showed a strong positive linear correlation with Ishak’s liver fibrosis scoring system [119]. Another study by Polasek et al. using rat and mice models showed that EP-3533-enhanced MR could distinguish fibrotic livers from control groups and there was positive correlation between EP-3533 gadolinium concentration and the Ishak scoring system (r=0.84(rats), r=0.79 (mice)). This was not the case with Gd-DTPA-enhanced MR [120]. A more recent study by Zhu et al using a rat model showed that combining the techniques of MR elastography and EP-3533 MR in a single exam provided an accurate means of staging hepatic fibrosis [121]. However, EP-3533 MR was retained in the bone and other tissues and the use of Gd-DTPA chelate carries the risk of gadolinium-associated nephrogenic systemic fibrosis [122].

#### CM-101:

CM101 is also another type I collagen T1 MR contrast agent which is more stable and not limited in terms of using Gadoterate meglumine chelate (Gd-DOTA). Christian et al. evaluated the biodistribution, metabolism, and pharmacokinetics of CM-101 in rats and mice models ex vivo and investigated the role of CM101 MR for detecting liver fibrosis in vivo using a 1.5T MR scan; the results were exciting. CM101 demonstrated fast blood clearance, whole-body elimination, and negligible accumulation in bone, kidneys, liver, and spleen [87].

#### RED (argubue-glycine-aspartic acid) probe targeting αvβ3:

With regard to the activation of HSCs in liver fibrogenesis, the integrin αvβ3 is expressed on HSCs, promotes HSCs adhesion and migration, and binds to ECM by means of a three amino acid sequence of RGD [123]. The RED peptide modified ultrasmall superparamagnetic iron oxide nanoparticle (RED-USPIO) is T2 MR contrast agent that specifically targets αvβ3 on activated HSCs. Zhang et al. intravenously administered RED-USPIO in the liver of rats with carbon tetrachloride-induced liver fibrosis. They found that the expression of αv and β3 on activated HSCs was upregulated and correlated well with the progression of liver fibrosis (r=0.954, 0.931, P<0.001, respectively) and the accumulation of iron particles in fibrotic liver specimens was significantly greater with RED-USPIO compared to the naked USPIO group [124]. Nuclear imaging techniques have been employed for acquiring molecular information in clinical applications and the results are exciting. Both nuclear medicine and molecular MRI can diagnosis and assess early stages of liver fibrosis with high sensitivity and accuracy in animal experiments. As the development of molecular imaging agents evolves, nuclear imaging using ECM-or HCS-specific probes may become valuable and promising techniques for assessing liver fibrosis. However, radioactivity is the inevitable limitation of nuclear medicine. Nevertheless, molecular MRI of liver fibrosis represents an effective additional method to manage this life-threatening disease.

## Conclusion

Liver fibrosis is a common pathological consequence of chronic liver disease and is characterized by the excessive accumulation of ECM components. HSCs are a key target in assessing liver fibrosis. Conventional MRI, US and CT can evaluate morphological and structural changes related to liver fibrosis and are useful in assessing fibrosis and cirrhosis. Functional imaging, including MRE, US elastography, and CT perfusion may be useful for detecting moderate to advanced liver fibrosis. Molecular imaging such as that used in nuclear medicine and molecular MRI may be valuable for detecting early liver fibrosis and monitoring the progression of disease. Although molecular imaging of liver fibrosis is still in a developmental phase, the concept of a target-specific molecular approach opens new avenues for effective management of this life-threatening disease.

## Figures and Tables

**Table 1:** Various modality staging options.

Methods	Modality	Early Stage (F0–1)	Moderate Stage (F2)	Severe Stage (F3–4)	References
	**MR**				
	Conventional MRI			+	[32]
	SWI			+	[33]
	DWI		+	+	[38–40]
	PWI		+	+	[44,45,48]
	MRE		+	+	[50,51]
	T1 mapping MRI		−	+	[56,59]
	MRI texture analysis		+	+	[61]
	Tip MR Imaging			+	[62,63]
	**US**				
	Conventional US			+	[65]
	Contrast-enhanced US			+	[67]
	US Elastography			+	[67]
	**SE**				
	TE (Fibroscan)		+		[68,69]
	ARFI		+		[71,72]
	SWE		+		[74]
	**CT**				
	Conventional CT			+	[77,78]
	CTP			+	[79,80]
	Fibro CT			+	[81]
	CT texture analysis			+	[82,83]
	Deep learning-based algorithms			+	[84,85]
Molecular Imaging	**Nuclear medicine**				
	(99m)Tc-3PRGD2 probe targeting integrin αvβ3		+		[93]
	(99m)Tc-GSA probe targeting Asialoglycoprotein		+		[95]
	99mTc-CBP1495 (CPKESCNLFVLKD) probe targeting type I collagen		+	+	[96]
	[18F]FEDAC probe targeting TSPO			+	[99]
	99mTc-MHS			+	[101]
	13N-NH3-H2OPET/CT		+	+	[102]
	4-[18F]fluoro-proline				[103]
	**Molecular MRI**				
	EP-3533 probe targeting type I collagen			+	[119–121]
	CM-101 probe targeting type I collagen		+		[87]
	RED probe targeting αvβ3			+	[124]
	+, reference provides staging of liver fibrosis.				
